# Antioxidant Activities of Hydrolysates of Arca Subcrenata Prepared with Three Proteases

**DOI:** 10.3390/md6040607

**Published:** 2008-11-20

**Authors:** Liyan Song, Tingfei Li, Rongmin Yu, Chunyan Yan, Shengfang Ren, Yu Zhao

**Affiliations:** 1 College of Pharmaceutical Sciences, Zhejiang University, Hangzhou 310058, China; 2 College of Pharmacy, Jinan University, Guangzhou 510632, China

**Keywords:** *Arca subcrenata* Lischke, hydrolysates, antioxidant activity, protease

## Abstract

In order to get products with antioxidant activity from *Arca subcrenata* Lischke, the optimal hydrolase and hydrolysis conditions were investigated in the paper. Three proteases (neutrase, alcalase and papain) were applied to hydrolyze the homogenate of *A. subcrenata*. An orthogonal design was used to optimize hydrolysis conditions, and the pH-stat methods was used to determine the degree of hydrolysis. Viewed from the angle of reducing power, such as scavenging activities against α,α-diphenyl-β-picrylhydrazyl (DPPH) radical and hydrogen peroxide, the antioxidant activities of the alcalase hydrolysate (AH) were superior to neutrase *h*ydrolysate (NH) and papain hydrolysate (PH), and its EC_50_ values in DPPH radical and hydrogen peroxide scavenging effect were 6.23 mg/ml and 19.09 mg/ml, respectively. Moreover, compared with products hydrolyzed by neutrase and papain, the molecular mass of AH was lower and its content of amino acid of peptides was higher. Therefore, alcalase was selected as the optimal enzyme to produce active ingredients since its hydrolysate exhibited the best antioxidant activity among them and possessed large amount of potential active peptides.

## 1. Introduction

In recent years, there is a growing interest to identify antioxidative properties in many natural sources including dietary proteins due to the potential health hazards of certain synthetic antioxidants [1]. Up to now, numerous products derived from hydrolyzed food proteins have been proven to possess noteworthy antioxidative activities against the peroxidation of lipids [2]. Several studies have proven that certain products from marine sources, such as prawn [3], tuna cooking juice [4], Alaska pollack frame [5] and skin [6] display significant antioxidant activities. Many antioxidant compounds, in particular peptides, were extracted by hydrolysis using various enzymes. The properties of functional peptides are highly influenced by their molecular masses and their structures, which are in turn greatly affected by processing conditions and the enzymes employed. Enzymatic hydrolysis, an effective approach for the release of bioactive peptides from protein sources, has become a valuable tool for changing and upgrading the protein nutrition and function [7].

*Arca subcrenata* Lischke, a marine animal native to the seas around China, has been used in treatments of tumor, anemia and inflammation for centuries in Chinese Traditional Medicines [8]. The concentration of protein and saccharide, proportions of amino acid, ultraviolet spectrum analysis and trace element analysis of this species had been previously reported [9]. Its hydrolysate was found to possess hypoglycemic activity on mice with alloxan-induced hyperglycemia, hypolipidic activity on experimental mouse model of hyperlipidemia [10]. However, no information is currently available on its potential antioxidant activity. During the course of our extensive screening program on marine traditional Chinese medicines for *in vitro* antioxidant activities, *A. subcrenata* was selected because of its widespread geographic distribution in China and its unique reputation in foods and folklore medicines [11].

In the present study, hydrolysis conditions of three enzymes, including neutrase, alcalase and papain, were optimized, the contents and compositions of amino acids in total hydrolysates and bioactive peptides were measured, and their antioxidant activities were determined. The work will be beneficial to the nutritional and medicinal utilization of this great marine resource.

## 2. Materials and methods

### 2.1 Materials

The frozen material of *A. subcrenata* was thawed to 4 °C and cleaned by distilled water. The defrosted material was diluted with double distilled water (1:2, w/v) and minced at 15,000 × *g* for 3 min by triturator. The homogenate was stored at –20 °C before use. Some homogenate was centrifuged at 22,000 × *g* for 20 min, and the supernatant was stored at –20 °C before use.

### 2.2 Enzymatic hydrolysis

Neutrase, alcalase and papain, with their proteolytic activities of 20 × 10^4^ U/g, were purchased from Nanning Pangbo Biological Engineering Co., LTD, China.

Hydrolysis experiments were carried out in a thermostatically stirred-batch reactor using the pH-stat method [12]. The pH value of homogenate of *A. subcrenata* was adjusted by adding 0.25 M NaOH. After adding each enzyme, the homogenate was constantly stirred at 500 × *g* for desired time at desired temperature. Calculation of degree of hydrolysis was based on the pH-stat method. Reactions were terminated by heating the solution to 90 °C for 15 min [13] to inactivate the enzyme. The resulting slurry was centrifuged at 22,000 *× g* for 20 min.

### 2.3 Optimization of hydrolysis conditions

An orthogonal design was employed to obtain the best combination of the critical parameters, e.g., temperature, pH, hydrolysis period, quantities of the enzyme and substrate (Table 1). The ranges of the parameters chosen for orthogonal design experiment were determined according to the instructions of the best enzyme activity and stability provided by Nanning Pangbo Biological Engineering Co., LTD, China.

### 2.4 Analytical methods

#### 2.4.1 Determination of the degree of hydrolysis

The following formula [14] was used to calculate value of degree of hydrolysis (DH) values:

$$\text{DH}\;(\%)\;=\;\;[B\;N_{b}/Mp\;\alpha \;h_{tot}]\;\;\times \;100$$

DH value is equal to the number of hydrolyzed peptide bonds divided by the number of peptide bonds in the substrate (*h*_tot_). The value of *h*_tot_ was estimated as the sum of the millimole of each individual amino acid per gram of protein (*N* × 6.25), this being found by determining the amino acid composition of the proteic substrate. Variable *B* was the amount of alkali consumed to keep the pH constant during the reaction. *N*_b_ was the normality of the alkali. *M*p was the mass of the substrate (protein, determined as *N* × 6.25) in the reaction, and α was the average degree of dissociation of α-NH_2_ groups released during hydrolysis.

#### 2.4.2 Determination of molecular mass distributions of hydrolysates

The method of Schagger [15] was employed with some modifications. By introducing 0.1 M Tricine as the trailing ion in the cathode buffer, authors succeeded in separating peptides with a MW down to about 1 kDa, which was impossible when glycine was used.

Electrophoresis was carried out in a BIO-RAD power PAC-300. The separation gel consisted of 2.06 ml of the solution of 40% T and 4% C, 2.86 ml of gel buffer, and 1.92 ml of 50% glycerol. Add water to final volume of 8 ml. Polymerize with 10% ammonium persulfate solution and TEMED (N, N, N′, N′-Tetramethylethylenediamine). A stacking gel of 2 cm height with 40% T, 4% C solution and gel buffer was used. Separation was carried out at a constant current of about 60 V for the first 40 min, then switch to 120 V and lasting for about 4 h until the Coomassie brilliant blue G250 band (added as a tracer) had reached the bottom of the gel. Gels were fixed for 1 h in 10% trichloroacetic acid (TCA), stained during 12 h in 2.5 g/L Coomassie brilliant blue R250. Destaining was done by keeping the gels for about 7 h in fresh destainer.

#### 2.4.3 Determination of antioxidant activities

*The assay of DPPH free radical scavenging activity:* The scavenging effect of hydrolysates on DPPH free radical was measured according to the method of Shimada [16] with some modifications. Each sample (2 ml) at varying concentrations was added to 0.5 ml of 0.2 mM ethanolic DPPH. After incubating for 30 min at the room temperature, the absorbance of the solution was measured at 517 nm with a spectrophotometer (UV2450 Shimadzu, Kyoto, Japan). The control was conducted in the same way except that distilled water was used instead of the sample. The blank was conducted in the same way except that distilled water was used instead of the ethanolic DPPH. The tests were performed in triplicate. Scavenging DPPH radical activity was calculated according to the following equation:

Scavenging effect(%) = {1−(S−SB)/ (C−CB)}  × 100

where S, SB, C and CB stand for the absorbance of the sample, the blank sample, the control and the blank control, respectively.

The assay of hydrogen peroxide scavenging activity: The effect of scavenging hydrogen peroxide (H_2_O_2_) was determined according to the method described by Ruch [17]. Four moles of hydrogen peroxide was prepared in 0.1 M phosphate buffer (pH 7.4) at 20 °C. The concentration of hydrogen peroxide was measured at 230 nm using the spectrophotometer. Different quantities of each sample were dissolved in 3.4 ml of 0.1 M phosphate buffer (pH 7.4), and then each mixed with 0.6 ml of hydrogen peroxide solution. Measurement of hydrogen peroxide concentration was conducted at 230 nm after reaction for 10 min. The blank sample solution was prepared as the above-mentioned sample solution without hydrogen peroxide. The control was conducted in the same way except that distilled water was used instead of the sample. The tests were performed in triplicate. Scavenging hydrogen peroxide activity was calculated according to the following equation:

Scavenging effect(%) = {1−(S−SB)/ (C−CB)}  × 100

where S, SB, C and CB stand for the absorbance of the sample, the blank sample, the control and the blank control, respectively.

*The assay of reducing power:* Determination of reducing power was conducted according to the method of Oyaizu [18]. Samples with different quantities (0, 10, 20, 30, 40, 50 and 60 mg) were added to 2.5 ml of 0.2 M phosphate buffer (pH 6.6) and 2.5 ml of 1% potassium ferricyanide. The mixture was incubated at 50 °C for 20 min. After adding 2.5 ml of 10% TCA, the mixture was centrifuged at 22,000 × *g* for 10 min, 2.5 ml supernatant was mixed with 2.5 ml of distilled water and 0.5 ml of 0.1% ferric chloride in a test tube. After reaction for 10 min, the solution absorbance was measured at 700 nm. Triplicate tests were conducted for each sample. The increase of absorbance for reaction mixture correlates directly to the increase of reducing power.

#### 2.4.4 Size exclusion chromatography

Molecular mass distribution of peptides in different hydrolysates was determined by gel permeation chromatography on a Sephadex G-25 column (1.0 cm × 90 cm; Amersham Pharmacia Biotech AB, Sweden), which was eluted with de-ionized water. Fractions in 5 ml each were collected at a flow rate of 0.5 ml/min, and the absorbance was measured at 280 nm, according to the procedure described by Tsai [19]. The standard curve of molecular mass demonstrated a linear correlation between the retention time and the logarithm of 200 – 2,000 Da peptides.

#### 2.4.5 Determination of free amino acids [20] and total amino acids of hydrolysates

The lyophilized hydrolysate (100 mg) was dissolved in 2 ml of de-ionized water. Mixed with 2 ml of 4.0% sulphosalicylic acid, the solution was centrifuged at 10,000 × *g* for 20 min at 4 °C to remove precipitate. The supernatant was used for analysis of free amino acids in the ICS-2500 ion exchange chromatography system.

One hundred milligrams of lyophilized hydrolysate were digested in 6 M HCl at 110 °C for 24 h [21], after which HCl was removed under vacuum. The dried samples were then reconstituted in 0.1 M de-ionized water. Subsequent amino acid analysis was carried out in the ICS-2500 ion exchange chromatography system.

The amount of peptides was equal to the difference in values between the amount of total amino acids and that of free amino acids.

#### 2.4.6 Statistical analysis

All the tests were done in triplicate and data were averaged. Standard deviation was also calculated. The SPSS statistic programme (Version 10.0) was used to evaluate significant differences (P < 0.05) between the means for each sample.

## 3. Results and Discussion

### 3.1 Optimization of enzymatic hydrolysis conditions

The enzymatic hydrolysis conditions were optimized using orthogonal design as described above. Based on the results and statistical analysis of orthogonal experiments (Table 1), the optimal hydrolysis conditions of three commercial enzymes, i.e., the hydrolysis temperature is 40, 45 and 55 °C, the hydrolysis time is 5, 4 and 6 h, E/S value is 6.0, 5.0 and 5.0%, pH is 7.5, 9.0 and 6.5 for neutrase, alcalase and papain respectively, were obtained. In addition, antioxidant activities of various hydrolysates prepared according to the orthogonal design were investigated. The results showed that the hydrolysis conditions were not significant factors influencing the antioxidant activity (data not shown). Therefore, we obtained the optimal conditions of enzymatic hydrolysis based on the DH value instead of the antioxidant activity.

As shown in Table 1, pH played the most important role in alcalase hydrolysation since the S value of alcalase for pH was the biggest. Similarly, E/S played the most important role in neutrase hydrolysation and papain hydrolysation [Table 1].

### 3.2 Effect of different enzymes on the degree of hydrolysis

The hydrolysis experiments were conducted under the optimal conditions of three enzymes in order to get DH values, which resulted from the above equation.

**Figure 1.**
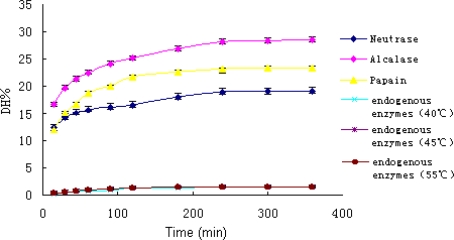
Hydrolytic curves of *A. subcrenata* by three commercial enzymes and the endogenous enzymes.

During the hydrolysis, the homogenate was rapidly converted from viscous mince into a free flowing liquid. Hydrolytic curves of *A. subcrenata* by the enzymes and endogenous enzymes alone were shown in Figure 1. All curves exhibited an initial fast reaction followed by a slowdown. The shape of these progress curves was similar to what has been reported for enzymatic hydrolysis of different protein substrates [22].

The DH values of the hydrolysates reflected the functions of both commercial enzymes and endogenous enzymes. Since the DH values of endogenous enzymes were less than 2%, the effect of endogenous enzymes was negligible. Therefore, the DH values overwhelmingly reflected the effect of commercial enzymes. In other words, the DH values represented the hydrolysis ability of essentially the three commercial enzymes.

### 3.3 Hydrolysate analysis by SDS-PAGE

SDS-PAGE was conducted in order to differentiate among the three hydrolysates. The result showed that alcalase and neutrase are relatively effective enzymes for hydrolysis compared with papain.

### 3.4 Molecular mass distributions of three hydrolysates

The gel permeation chromatography on a Sephadex G-25 column was used to study molecular mass distributions of different hydrolysates. The chromatogram indicated that three hydrolysates of *A. subcrenata* were made up of peptides with low molecular masses. AH yielded a large quantity of small peptides, whose molecular masses were below 2,126 Da, while those by PH turned out to be above 2,126 Da. The molecular mass distribution reveals that NH has less small peptides than the other two, which is consistent with the results from the three enzymes’ hydrolysis degrees.

### 3.5 Antioxidant activities of hydrolysates

DPPH radicals, stable in ethanol, show maximum absorbance at 517 nm. Once DPPH radicals meet with a proton-donating substance such as antioxidants, they are scavenged, resulting in decrease of their absorbance. The above method can be used for characterization of hydrolysate with free radical scavenging activity [23].

**Figure 2.**
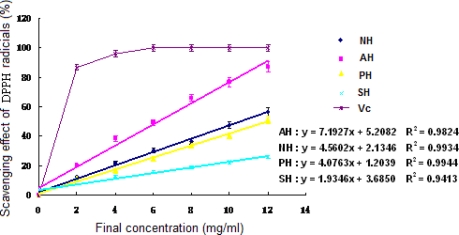
**DPPH free radical scavenging activity of the extracts and hydrolysates of** ***A. subcrenata.*** NH: the hydrolysate treated by neutrase; AH: the hydrolysate treated by alcalase; PH: the hydrolysate treated by papain; SH: the supernatant of unprocessed homogenate of *A. subcrenata*. Ascorbic acid (Vc) was used as positive control. Regression equations were obtained from linear regression of the concentrations of the extracts and hydrolysates of *A. subcrenata* and DPPH radical scavenging effects. Each value is expressed as mean ± S.D. (*n* = 3).

Seen from Figure 2, all three hydrolysates exhibited the ability to eliminate DPPH radicals. Furthermore, the DPPH radical scavenging activity could be enhanced with the increase of protein concentrations in the samples (P < 0.05). AH showed a better effect than the other two in the range from 2 to 12 mg/ml (P < 0.05). According to the regression equations, the EC_50_ values of the hydrolysates for scavenging activity against DPPH were 10.50 mg/ml (NH), 6.23 mg/ml (AH) in order.

The results revealed that the supernatant of *A. subcrenata* and its hydrolysates possibly contained substances that act as electron donors, convert free radicals to more stable products and terminate the radical chain reaction.

Hydrogen peroxide forms *in vivo* under the setting of many oxidizing enzymes, such as superoxide dismutase. Together with reactive oxygen species (ROS), hydrogen peroxide can damage several cellular components. Hydrogen peroxide is a relatively unstable metabolic product responsible for the generation of hydroxyl radicals and singlet oxygen from Fenton reaction. Hydroxyl radicals and singlet oxygen can initiate lipid peroxidation and be toxic to cells. Therefore, it is crucial for cells to remove hydrogen peroxide as an antioxidant defense [24].

**Figure 3.**
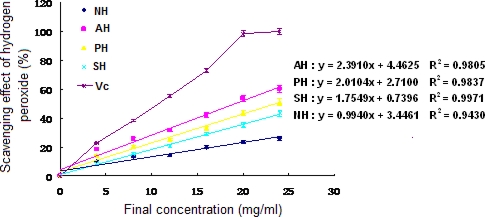
**Hydrogen peroxide scavenging activity of the extracts and hydrolysates of** ***A. subcrenata.*** NH: the hydrolysate treated by neutrase; AH: the hydrolysate treated by alcalase; PH: the hydrolysate treated by papain; SH: the supernatant of unprocessed homogenate of *A. subcrenata*. Ascorbic acid (Vc) was used as positive control. Regression equations were obtained from linear regression of the concentrations of the extracts and hydrolysates of *A. subcrenata* and hydrogen peroxide scavenging effects. Each value is expressed as mean ± S.D. (*n* = 3).

The dose-dependent antioxidant effect (P < 0.05) was observed from hydrogen peroxide scavenging activity of both supernatant and hydrolysates of *A. subcrenata* (Figure 3). The EC_50_ value of AH was 19.09 mg/ml, which displayed the highest scavenging effect on hydrogen peroxide among all three hydrolysates (P < 0.05), while that of PH was 23.52 mg/ml. Notably, NH at the final concentration of 24 mg/ml exhibited 26.11% scavenging effect on hydrogen peroxide, which was lower than the supernatant of *A. subcrenata*. It may be deduced that neutrase could destroy or decompose the supernatant function of scavenging hydrogen peroxide.

**Figure 4.**
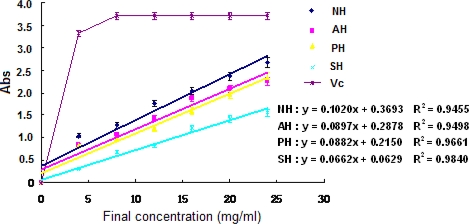
Reducing power of the extracts and hydrolysates of *A. subcrenata.*

The experiment of Fe^3+^– Fe^2+^ transformation in the presence of the supernatant and the hydrolysates of *A. subcrenata* was done using the method of Oyaizu [18] for the assay of the reductive ability. The reducing powers of the hydrolysates were excellent and concentration-dependent (P < 0.05) (Figure 4). NH showed the highest reducing power and AH came in second (P < 0.05), which was different from above-mentioned results of DPPH radical and hydrogen peroxide scavenging effect.

On the whole, the hydrolysates exhibited apparent antioxidant activities, implying that the hydrolysates seemed to contain some antioxidant substances.

### 3.6 Determination of amino acid composition of hydrolysates

The amino acid compositions of the hydrolysates and the supernatant of *A. subcrenata* were analyzed, and the results were shown in Table 2. The compositions of total amino acids of the supernatant and the hydrolysates of *A. subcrenata* were similar, whose major amino acids were Gly, Leu, Gln and Asn. It indicated that the hydrolysate treated with alcalase had more soluble peptides which could not be removed by centrifuging. Notably, AH had the highest DH among the enzyme hydrolysates. This result is consistent with the findings of Gbogouri [25], who reported that hydrolysates had an excellent solubility at high DH.

During hydrolysis, a wide variety of smaller peptides and free amino acids were generated, depending on enzyme specificity. Changes in size, level and composition of free amino acids and small peptides could affect the antioxidant activity [26], and this might lead to the differences in antioxidant activity of the three hydrolysates of *A. subcrenata*.

Jun [27] reported that yellowfin sole hydrolysate, treated at lowest DH (22%), had a higher antioxidant activity than high DH. This result was not consistent with the analyses of AH, which had the highest DH and antioxidant activity. However, data had shown that most active peptides were of low molecular mass [28]. These small peptides have relevant biological activity *in vivo* and were more easily absorbed in the intestinal tract than larger molecules. As shown in Table 2, many small peptides were obtained in AH; therefore it is assumed that AH would possess more potential in producing biologically active peptides. NH was rich in free amino acid seen from Table 2, but the content of amino acid of peptides was less than that of the others. The data implies that the main effect of neutrase was producing amino acids from *A. subcrenata*, which was not useful in generating bioactive peptides.

As shown in Table 2, the amount of Thr, Val, His, Phe and Tyr of AH was higher than that of PH and NH. Some amino acids, such as His, Tyr, Met and Cys, have shown obvious antioxidant activity [29]. The N-terminal histidine could contribute higher antioxidant activity to the peptides [30]. The activity of carnosine, a well-known antioxidant peptide, had been attributed at least in part to the presence of key His residues [31]. Some antioxidant peptides were from digests of a soybean protein, including hydrophobic amino acids, Val or Leu, at the N-terminal positions, and Pro, His, or Tyr in the sequences [32]. Therefore, the higher antioxidant activities of AH seemed to be caused by these amino acids in the hydrolysate peptides. It was presumed that the amino acids such as His, Val and Thr presented in the sequence of the hydrolysate peptides had favored the radical scavenging and reductive abilities. The total content of hydrophobic amino acids of AH was higher than that of the others. For peptides, an increase in hydrophobicity would increase their solubility in lipid and therefore enhanced their antioxidant activities [33].

## 4. Conclusion

Three enzymes (neutrase, alcalase and papain), were applied to hydrolyze the materials of *A. subcrenata* in this research. By an orthogonal design, the optimal enzymatic hydrolysis conditions were obtained. All three hydrolysates exhibited strong reducing power as well as DPPH radicals and hydrogen peroxide scavenging activities. A large amount of peptides, whose molecular masses were lower than 2,126 Da, were measured in AH. Furthermore, this fraction was superior to the other two hydrolysates in terms of antioxidant activities. Thus, alcalase was the optimal enzyme for *A. subcrenata* to produce the active ingredients. AH could be potential antioxidant for use in foods, dietary supplements and medicines. Further detailed study on the relationship of antioxidant activity and peptides, and the mechanism of antioxidant activity of AH of *A. subcrenata* is currently under progress.

## Figures and Tables

**Table 1.** Results and analysis of orthogonal experiment of three proteases

No	Variable														
	Temperature (°C)			Time (h)			E/S (%)			pH			DH (%)		
	N	A	P	N	A	P	N	A	P	N	A	P	N	A	P
1	1(40)	1(45)	1(50)	1(4)	1(4)	1(4)	1(4.0)	1(3.0)	1(3.0)	1(6.5)	1(8.0)	1(6.5)	13.00	17.20	8.10
2	1	1	1	2(5)	2(5)	2(5)	2(5.0)	2(4.0)	2(4.0)	2(7.0)	2(8.5)	2(7.0)	13.80	16.10	3.90
3	1	1	1	3(6)	3(6)	3(6)	3(6.0)	3(5.0)	3(5.0)	3(7.5)	3(9.0)	3(7.5)	19.40	23.70	7.70
4	2(45)	2(50)	2(55)	1	1	1	2	2	2	3	3	3	12.00	19.70	5.40
5	2	2	2	2	2	2	3	3	3	1	1	1	14.30	12.90	12.20
6	2	2	2	3	3	3	1	1	1	2	2	2	12.50	6.40	7.80
7	3(50)	3(55)	3(60)	1	1	1	3	3	3	2	2	2	16.20	16.10	9.80
8	3	3	3	2	2	2	1	1	1	3	3	3	17.30	17.80	5.20
9	3	3	3	3	3	3	2	2	2	1	1	1	9.10	9.20	8.10
^K^1	46.20	57.00	19.70	41.20	53.00	23.30	42.80	41.40	21.10	36.40	39.30	28.40			
^K^2	38.80	39.00	25.40	45.40	46.80	21.30	34.90	45.00	17.40	42.50	38.60	21.50			
^K^3	42.60	43.10	23.10	41.00	39.3	23.6	49.90	52.70	29.70	48.70	61.20	18.30			
^R^1	15.40	19.00	6.57	13.73	17.67	7.77	14.27	13.80	7.03	12.13	13.10	9.47			
^R^2	12.93	13.00	8.47	15.13	15.6	7.10	11.63	15.00	5.80	14.17	12.87	7.17			
^R^3	14.20	14.37	7.70	13.67	13.1	7.87	16.63	17.57	9.90	16.23	20.40	6.10			
S	3.05	19.77	1.83	1.36	10.47	0.35	12.51	7.42	8.85	8.41	36.68	5.93			

Note: Ki represents the sum of DH at level i (%); Ri represents the average of DH at level i (%); S represents the sum of deviation squares (10**^–^**^4^).

**Table 2.** The contents (mmol/L) of amino acids of *A. subcrenata* extracted from various fractions

Amino acid	Supernatant	NH	AH	PH
		T^a^	F^b^	AAP^c^	T^a^	F^b^	AAP^c^	T^a^	F^b^	AAP^c^
Arg	19.38	20.79	14.53	6.26	23.56	11.93	11.64	19.40	9.85	9.48
Lys	10.67	16.04	4.82	11.22	24.31	3.42	20.89	20.13	2.92	17.21
Ala^d^	15.87	16.32	9.70	6.62	27.94	8.74	19.20	20.65	1.30	19.35
Thr	10.88	12.01	9.38	2.63	22.19	13.77	8.42	15.89	11.25	4.64
Gly	26.22	26.68	4.37	22.31	32.97	17.17	15.80	26.28	3.15	20.13
Val^d^	7.88	11.18	6.73	4.46	18.32	-	18.32	11.81	2.06	9.75
Ser	11.47	12.85	-	12.88	27.80	-	27.80	16.23	-	16.23
Pro^d^	5.48	7.60	5.78	1.82	9.18	4.43	4.75	11.08	2.31	8.77
Ile^d^	5.90	7.68	5.42	2.26	14.84	6.12	8.72	10.69	2.77	7.92
Leu^d^	16.31	22.34	10.85	11.50	37.58	5.80	31.78	28.53	3.96	24.57
Met^d^	4.09	6.16	3.87	2.29	11.26	2.75	8.51	8.04	1.74	6.29
His	3.15	4.09	3.83	0.26	6.61	0.85	5.76	5.03	3.68	1.35
Phe^d^	5.46	6.64	3.54	3.09	11.60	-	11.60	6.56	2.99	3.57
Gln	34.26	42.04	7.18	34.87	66.05	6.15	59.90	50.41	2.99	47.42
Asn	19.56	26.31	1.40	24.91	40.99	0.64	40.36	33.03	0.75	32.28
Cys	0.23	0.96	0.11	0.84	1.15	-	1.15	0.68	-	0.68
Tyr	3.61	3.77	0.33	3.44	8.82	2.61	6.22	5.24	1.95	3.29
Total	200.42	243.46	91.84	151.66	385.17	84.38	300.82	289.68	53.67	232.93

a: total AA of the supernatant and the hydrolysates;

b: free AA of hydrolysates;

c: AA from peptides of the hydrolysates (The content of AA of peptide = the content of total AA – the content of free AA);

d: The hydrophobic amino acids have been marked by black underline.
